# Removal of gut symbiotic bacteria negatively affects life history traits of the shield bug, *Graphosoma lineatum*

**DOI:** 10.1002/ece3.7188

**Published:** 2021-02-15

**Authors:** Naeime Karamipour, Yaghoub Fathipour, Mohammad Mehrabadi

**Affiliations:** ^1^ Department of Entomology Faculty of Agriculture Tarbiat Modares University Tehran Iran

**Keywords:** Gammaproteobacteria, gut bacteria, host, *Pantoea*‐like symbiont, symbiont interaction

## Abstract

The shield bug, *Graphosoma lineatum* (Heteroptera, Pentatomidae), harbors extracellular *Pantoea*‐like symbiont in the enclosed crypts of the midgut. The symbiotic bacteria are essential for normal longevity and fecundity of this insect. In this study, life table analysis was used to assess the biological importance of the gut symbiont in *G. lineatum*. Considering vertical transmission of the bacterial symbiont through the egg surface contamination, we used surface sterilization of the eggs to remove the symbiont. The symbiont population was decreased in the newborn nymphs hatched from the surface‐sterilized eggs (the aposymbiotic insects), and this reduction imposed strongly negative effects on the insect host. We found significant differences in most life table parameters between the symbiotic insects and the aposymbiotics. The intrinsic rate of increase in the control insects (0.080 ± 0.003 day^−1^) was higher than the aposymbiotic insects (0.045 ± 0.007 day^−1^). Also, the net reproductive and gross reproductive rates were decreased in the aposymbiotic insects (i.e., 20.770 ± 8.992 and 65.649 ± 27.654 offspring/individual, respectively), compared with the symbiotic insects (i.e., 115.878 ± 21.624 and 165.692 ± 29.058 offspring/individual, respectively). These results clearly show biological importance of the symbiont in *G. lineatum*.

## Graphical abstract

In this study, life table analysis was used to assess the biological importance of the gut symbiont in the shield bug, *Graphosoma lineatum* (Heteroptera, Pentatomidae). Surface sterilization of the eggs was used to remove the symbiont. The symbiont population was decreased in the newborn nymphs hatched from the surface‐sterilized eggs (the aposymbiotic insects), and this reduction imposed strongly negative effects on the life history traits of the insect host. We found significant differences in most life table parameters between the symbiotic insects and the aposymbiotics.

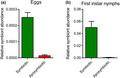

## 1 INTRODUCTION

Symbiotic associations between insects and microorganisms are among the key features that have enabled insects to occupy a wide range of ecological niches (Brownlie & Johnson, 2009). In this relationship, insects provide suitable condition for microbial symbionts and in many cases transmit them through the generation by vertical and horizontal transmission. Instead, the symbionts mainly conferred to the host any benefit that increases the survival and fecundity of the host. Some of endosymbionts are obligate for survival of their host such as *Buchnera* in aphids and *Wigglesworth* in tsetse fly. These symbionts provide essential nutrients like essential amino acids and vitamins for their hosts (Baumann, 2005; Buchner, 1965; Wernegreen, 2002). Elimination of these symbionts often has strong negative effect on their insect hosts including retarded growth and significant reduction in survival rates and fecundity (Abe et al., 1995; Aksoy, 1995; Hosokawa et al., 2006; Kikuchi et al., 2009; Zhang et al., 2015). In addition to obligate endosymbionts, facultative symbionts are also found in insects. Insects can live without their facultative symbionts; however, these symbionts often provide fitness advantages for their host or in some cases they are reproductive parasites (Ayoubi et al., 2020; Bagheri et al., 2019a, 2019b; Brownlie & Johnson, 2009).

Insects in suborder of Heteroptera including the shield bug *Graphosoma lineatum* (Pentatomidae) harbor extracellular endosymbiont in the midgut. The symbiont occupy sac‐like or tubular crypts that derived from the forth section of the midgut and play essential role in their host (Hayashi et al., 2015; Hosokawa et al., 2006; Kashkouli et al., 2019; Kikuchi et al., 2009; Mehrabadi et al., 2012). Symbionts of stinkbugs mainly belong to a distinct lineage of the Gammaproteobacteria (Fukatsu & Hosokawa, 2002; Hayashi et al., 2015; Kaiwa et al., 2011; Kashkouli et al., 2020; Kikuchi et al., 2009; Matsuura et al., 2014). These plant‐sucking insects transmit the beneficial bacterial endosymbiont through the generation (vertical transmission) by egg smearing and newly hatched nymphs probing outer egg surfaces to receive symbionts (Fukatsu & Hosokawa, 2002; Hayashi et al., 2015; Kikuchi et al., 2009). The vertical transmission indicates these symbiotic microorganisms play important biological functions in their hosts, so that elimination of them from the host mainly make retarded growth and elevated mortality (Bistolas et al., 2015; Hayashi et al., 2015; Karamipour et al., 2016; Kashkouli et al., 2019).

The endosymbionts affect different aspect of their host from biology and physiology to ecology and behavior. Often studies on symbionts restricted to the impact of them on their host, while the symbionts can influence on interaction of their partners with other species in the community level (Ferrari & Vavre, 2011). Symbionts, for example, can make dramatic change in population of their host in the field (Luo et al., 2017). Therefore, it seems that study on dynamic population changes that cause by endosymbiont is necessary. For example, studies on aphides indicated *Regiella* secondary endosymbionts can change life history of their host (Da et al., 2016; Luo et al., 2017). Also, symbiont elimination in *Nezara viridula* affects the life history of their hosts (Prado et al., 2009).

Life table analyses are the most widespread methods to study life history traits and population dynamic (Khanamani et al., 2013; Luo et al., 2017). This analytic tool is important to prediction of population growth and can clarify the impact of symbionts on the population features. For instance, the intrinsic rate of increase, which is most important parameters in life table analysis, indicates the reproductive potential of population (Luo et al., 2017).

There are two methods for life table analysis including female age‐specific life table and age‐stage, two‐sex life table. In the first method, the impact of males and also variation in development rate, which make stage differentiation, on life table parameters has been ignored. In contrast, age‐stage, two‐sex life table is incorporating impact of both sexes and developmental rates variation that occur between individuals. Therefore, age‐stage, two‐sex life table makes more realistic data from different aspects of insect biology and has been widely used in many studies (Chi & Liu, 1985).

It has been reported that *G. lineatum* harbors a *Pantoea*‐like primary symbiont in the fourth section of the midgut (PLSGL: *Pantoea*‐like primary symbiont of *G. lineatum*). These insects transmit PLSGL to the next generation by contamination of the eggs surface (Karamipour et al., 2016). In the present study, the effect of PLSGL on life history traits was evaluated to find out the biological importance of this gut symbiont in *G. lineatum*. Our results indicate that reduction of PLSGL negatively affects life history traits of the *G. lineatum*. These results confirm the contribution of the gut symbiont in the biology of *G. lineatum*.

## 2 MATERIAL AND METHODS

### 2.1 Insects

We established a laboratory colony of *G. lineatum* from populations that received from Agriculture and Natural Resources Research Center of Varamin, Iran. Insects were reared in growth chamber at 27 ± 1°C, 60 ± 5% RH, and a photoperiod of 16:8 hr condition in the laboratory. The seeds of Fennel, *Foeniculum vulgare*, and wet small ball of cottons were used to feed *G. lineatum*.

### 2.2 Symbiont removal

To remove PLSGL, we used egg surface sterilization by hypochlorite sodium as described before (Karamipour et al., 2016). To do this, the egg surface of *G. lineatum* was sterilized by dipping in 70% ethanol for 10 min, followed by 10% hypochlorite sodium for 15 s and washing with 70% ethanol. The control egg masses were treated similarly but distilled water was used instead of 10% hypochlorite sodium.

### 2.3 DNA extraction and real‐time PCR (qPCR)

qPCR was used to confirm if the symbiont is successfully removed. To do this, DNA extraction was performed from the surface‐sterilized eggs, the control eggs, the aposymbiotic first‐instar nymphs, and the control first instars of *G. lineatum*. DNA was extracted from 10 first‐instar nymphs and 10 egg masses as described before (Glatz et al., 2003). Moreover to assess quality of the extracted DNA, 5 μl of DNA samples was ran on 1% agarose gel. Concentrations of DNA samples were also quantified and adjusted to the same concentration. The specific primers including Glsym‐groEl‐F (TTTCTAACGCCGGTGAAGAG) and Glsym‐groEl‐R (ACCGAAGTCGATCATGTTGC) for quantifying the bacterial population and penta‐18srRNA‐F (CCTGCGGCTTAATTTGACTC) and penta‐18srRNA‐R (AACTAAGAACGGCCATGCAC) primers targeting *18S rRNA* gene as the reference gene were used for qPCR analysis (Karamipour et al., 2016). The qPCR performed by SYBR green (Ampliqon) with a micPCR instrument (BMS). The temperature profile was 95°C for 15 min, followed by 40 cycles of 95°C for 15 s, 15 s at the annealing temperature, and 72°C for 20 s. qPCR data were analyzed using the ΔΔ*C*_t_ method (Livak & Schmittgen, 2001).

### 2.4 Life table parameters

We utilized the age‐stage, two‐sex life table theory to determine the life table parameters (Chi, 1988; Chi & Liu, 1985). The age‐stage‐specific survival rate (*s_xj_*) (the chance of the survival of a newborn nymph to the age *x* and develop to stage *j*); the age‐stage‐specific fecundity (*f_xj_*) (mean number of eggs produced by females of the age *x* and stage *j*); the age‐specific survival rate (*l_x_*) (the probability of survivorship of a newborn nymph to reach the age *x*); the age‐specific fecundity (*m_x_*) (mean number of eggs produced per individual of the age *x*); and the population growth parameters including the intrinsic rate of increase (*r*) (the rate of increase in population); the finite rate of increase (*λ*) (the number of times the population multiplies per day); the gross reproductive rate (*GRR*) (the total eggs produced per individual per generation without considering the survivorship of the individuals); and the net reproductive rate (*R*_0_) (the total eggs produced per individual per generation with considering the survivorship of the individuals) and the mean generation time (*T*) (the time that a population requires to increase its size to the *R*_0_‐fold) are calculated as mentioned in the literature (Chi & Liu, 1985; Fathipour & Maleknia, 2016). The equations that utilized for calculation of female and two‐sex life table parameters and difference between them are shown in Table 1 (Fathipour & Maleknia, 2016). In addition, the proportion of survivorship of a newborn nymph to achieve age *x* (*l_x_*) was statistically compared between the symbiotic and aposymbiotic insects. This was performed using the two proportion test to check if the proportion of the probability of survivorship of a newborn nymph that achieve age *x* in symbiotic insects are the same as this proportion in aposymbiotic insects.

**TABLE 1:** Mean duration of different stages (±*SE*) (in days) of *Graphosoma lineatum* in aposymbiotic and symbiotic insects

Stages	Treatment	Mean duration of different stages (day)	*t*	*df*	*p*
Nymph 1	Symbiotic	3.2909 ± 0.06	1.679	1, 101	.094
	Aposymbiotic	3.1458 ± 0.05			
Nymph 2	Symbiotic	3.6000 ± 0.17	−6.804	1, 79	.000
	Aposymbiotic	6.1613 ± 0.38			
Nymph 3	Symbiotic	3.7347 ± 0.08	−5.214	1, 77	.000
	Aposymbiotic	5.1000 ± 0.30			
Nymph 4	Symbiotic	4.2340 ± 0.07	−2.843	1, 73	.005
	Aposymbiotic	4.6071 ± 0.10			
Nymph 5	Symbiotic	6.6304 ± 0.09	−5.200	1, 68	.005
	Aposymbiotic	7.5417 ± 0.14			
Female	Symbiotic	78.7143 ± 4.01	1.797	1, 26	.088
	Aposymbiotic	64.5714 ± 6.77			
Male	Symbiotic	82.2800 ± 4.03	3.362	1, 40	.005
	Aposymbiotic	54.1765 ± 8.25			

To obtain the biological parameters, females that were belonging to the same colony were allowed to lay eggs for 24 hr and these eggs were used to start the experiments. Sixty eggs of the same age (as a cohort) were selected from the main colony for each treatment. The nymphs were individually isolated in a separate plastic box from second nymph because the first nymphs have aggregation behavior. The data were daily recorded until the death of the last individual of the cohort.

The adult pre‐ovipositional period (APOP, the duration from female adult emergence to the first oviposition), total pre‐ovipositional period (TPOP, the duration from egg to the first oviposition), and mean duration of different stages were compared by *t* test using SPSS (IBM SPSS Inc.). Also, the proportion of survivorship of a newborn nymph to achieve age *x* (*l_x_*) was estimated using Minitab 16 software (Minitab). To compute the population parameters (*r*, *λ*, *GRR*, *R*_0_, and *T*), we utilized the TWOSEX‐MSChart program (Chi, 2016). Moreover, the standard errors of the population parameters were calculated by using the bootstrap procedure with 40,000 resampling. The means of the bootstrap pseudovalues of the population parameters were compared using the paired bootstrap method.

The parameters like APOP, TPOP, and mean duration of different stages have real values that are derived from different replications. Therefore, comparison of these parameters can be performed using *t* test analysis. Whereas, for comparison of the life table parameters, we have to make many pseudovalues using bootstrap resampling. Therefore, we have the huge numbers of the pseudovalues of the life table parameter (e.g., 40,000 here) that have been artificially generated and we have to use the paired bootstrap rather than *t* test analysis for comparison of the life table parameters (see Bahari et al., 2018 for details).

## 3 RESULT

### 3.1 Symbiont removal

**FIGURE 1:**
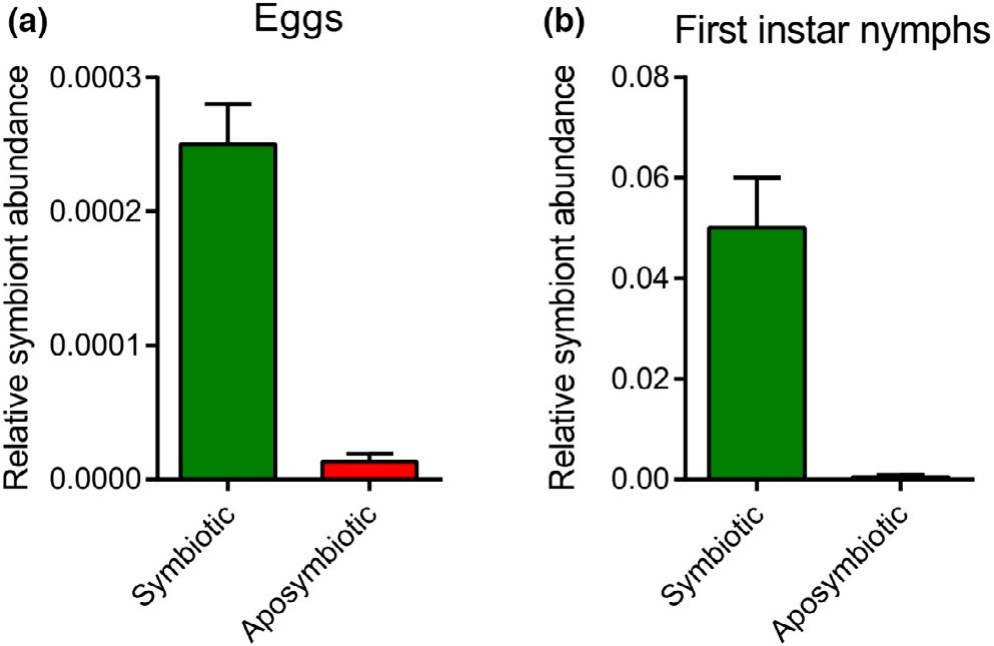
Egg surface sterilization for elimination of bacterial symbionts. (a) QPCR analysis of the control eggs and surface‐sterilized eggs of *Graphosoma lineatum*. (b) QPCR analysis of bacterial symbiont in first nymphs of *G. lineatum* emerged from the control and surface‐sterilized eggs (Karamipour et al., 2016). The egg sterilization strongly decreased bacterial symbiont intensity in the treated eggs and the aposymbiotic nymphs in comparison with the controls. ***p < .001, *t* test. *N* = 10

The egg sterilization by 10% hypochlorite sodium for 15 s had no effect on the rate of egg hatching; also, the longevities of the first nymphs were similar in the control and the aposymbiotic insects (Table 1). These results indicated that hypochlorite sodium had no negative effect on the rate of nymph emergence. QPCR analyses also showed severe decrease in population of PLSGL in the surface‐sterilized eggs relative to the control eggs (Figure 1a), as well as in the aposymbiotic first nymphs compared with the symbiotics (Figure 1b) (Karamipour et al., 2016).

### 3.2 Adult and total pre‐ovipositional period

**FIGURE 2:**
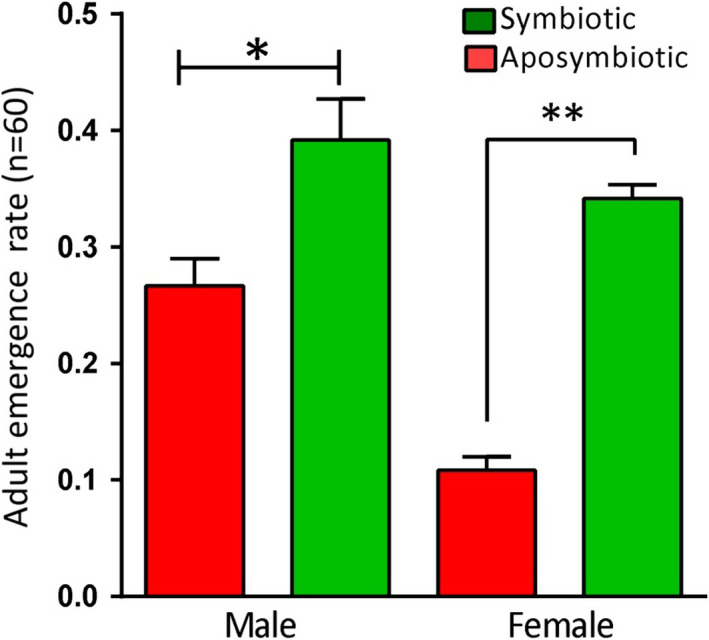
The emergence rates of males and females from the control and surface‐sterilized eggs. Removal of the symbionts decreased female and male emergence. *n* = number of individuals *p < .05; **p < .01

The APOP had no significant difference in the control (16.57 ± 3.48 days) and the aposymbiotic insects (11.61 ± 0.42 days). Nonetheless, the TPOP was significantly different between the two experimental groups; the aposymbiotic group showed longer TPOP in comparison with the symbiotic insects (49.28 ± 3.48 and 39.85 ± 0.41 days in control and aposymbiotic insects, respectively) (Table 2). The presence of the symbiont significantly affected the duration of different life stages of *G. lineatum* (Table 1). According to the results, duration of nymphal stages except first‐instar nymph was significantly longer in aposymbiotic insects. Moreover, the emergence of female in the control insects was higher than of the aposymbiotics (Figure 2).

**TABLE 2:** APOP and TPOP parameters of *Graphosoma lineatum* in the symbiotic and the aposymbiotic insects

Parameter	Aposymbiotics	Symbiotics	*t*	*df*	*p* < .05
APOP (day)	16.57 ± 3.48^a^	11.61 ± 0.42^a^	−2.393	1, 26	.20
TPOP (day)	49.28 ± 3.48^b^	39.85 ± 0.41^a^	−4.558	1, 26	.035

Note

The means followed by the same letter in each row are not significantly different (*p* < .05, *t* test).

Abbreviations: APOP, distance between female adult emergence and first oviposition; TPOP, distance between the egg and the first oviposition.

### 3.3 Age‐stage‐specific survival rate (*s_xj_*)

**FIGURE 3:**
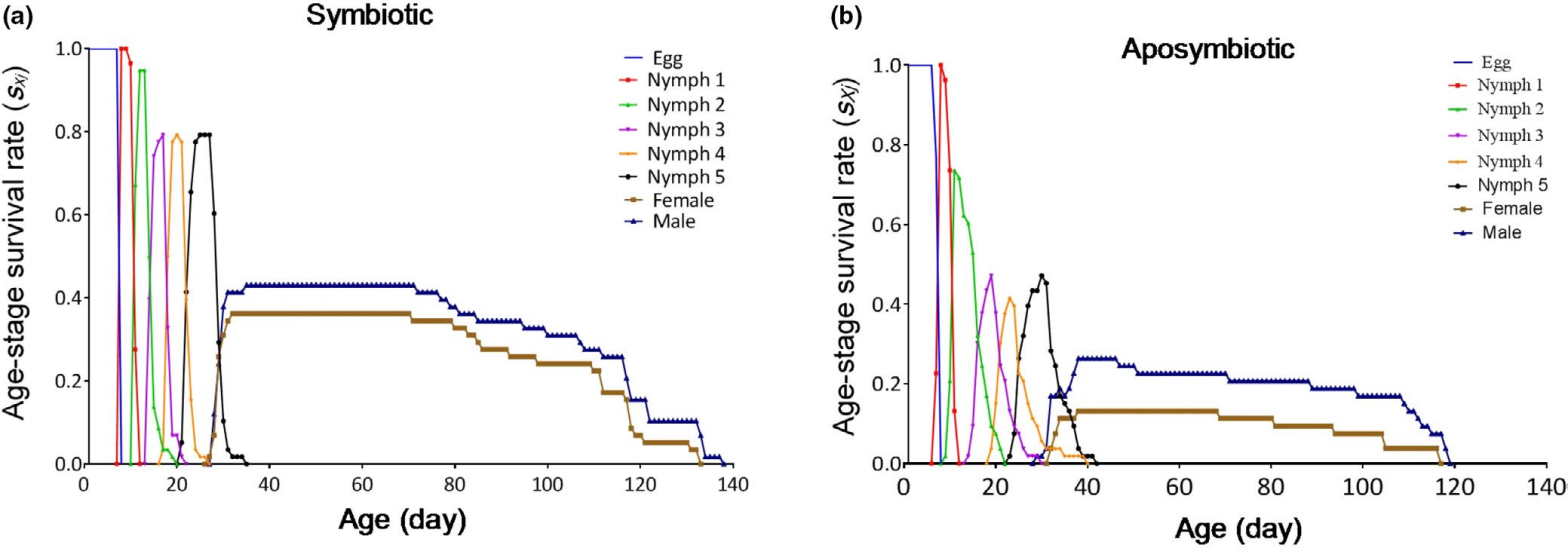
Age‐stage survival rate (*s_xj_*) of *Graphosoma lineatum* using the age‐stage, two‐sex life table. (a) Age‐stage survival rate of symbiotic insects. (b) Age‐stage survival rate of aposymbiotics

The posi to survival of a newborn nymph to each age stage of *G. lineatum* in the symbiotic and the aposymbiotic insects are shown in Figure 3. This curve shows the survival rate and stage differentiation. In addition, overlaps between different stages also are shown in the curves of *s_xj_*. Since there are variable development rates between individuals, overlaps among stages can be seen in this curve.

### 3.4 Age‐specific survivorship, age, and age‐stage‐specific fecundity

**FIGURE 4:**
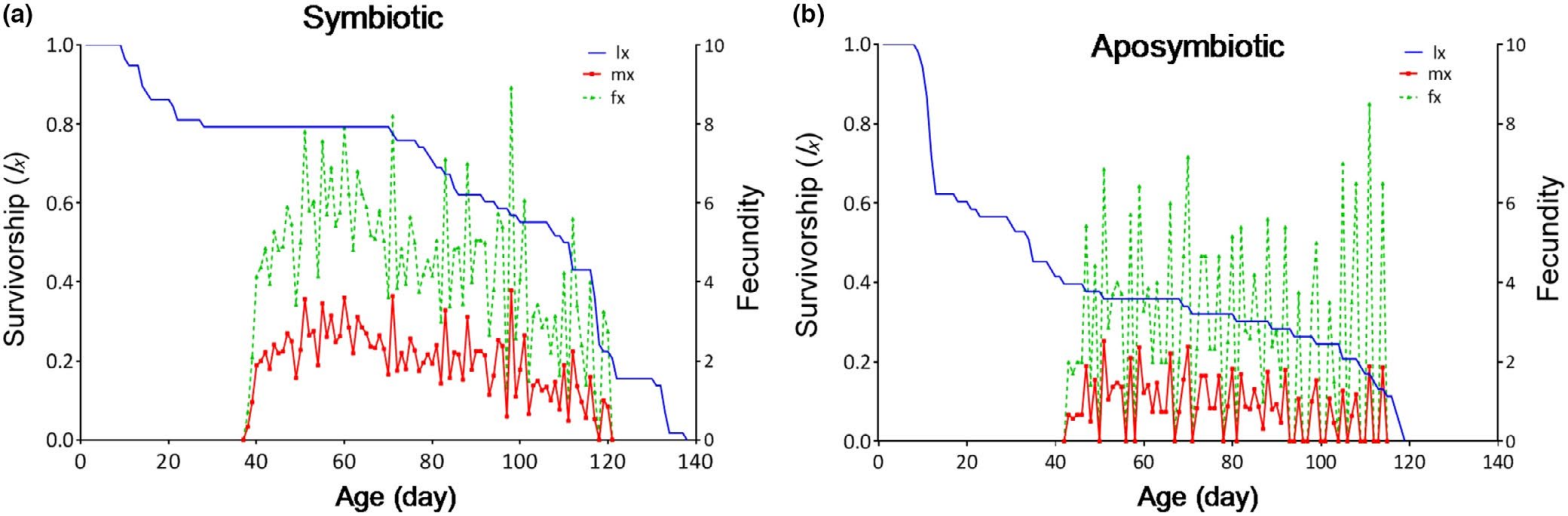
Age‐specific survivorship (*l_x_*), age‐stage fecundity of female (*f_xj_*), and age‐specific fecundity (*m_x_*) of *Graphosoma lineatum* in the symbiotic (a) and the aposymbiotic (b) insects, using the age‐stage, two‐sex life table

The age‐specific survivorship (*l_x_*), age‐specific fecundity (*m_x_*) of the aposymbiotic, and age‐stage‐specific fecundity (*f_xj_*) of the control insects are shown in Figure 4. According to the *f_xj_* curve, the oviposition of the first female happened at the age of 37 and 42 days on the control and aposymbiotic insects, respectively. Furthermore, the *m_x_* curve showed the highest daily fecundity of 3.7 and 2.5 eggs that occurred at the age of 97 and 50 days in the symbiotic and the aposymbiotic insects, respectively. Moreover, the curve of *l_x_* indicates that the probability of survivorship decrease with age increasing (Figure 4a,b). According to our result, the first significant effect of symbiont elimination occurred at third‐instar nymph where the number of living individual at the beginning of this stage in aposymbiotic insects was significantly decreased in comparison with symbiotic insects (0.868852 and 0.573770 in symbiotic and aposymbiotic insects, respectively) (*p* < .000; Table 3).

**TABLE 3:** The proportion of survivorship of a newborn nymph to achieve age *x* (*l_x_*) of *Graphosoma lineatum* in aposymbiotic and symbiotic insects

Age *x*	Treatment	*l_x_*	*p*
Nymph 1	Symbiotic	1.00	.313
	Aposymbiotic	0.98	
Nymph 2	Symbiotic	0.93	.138
	Aposymbiotic	0.85	
Nymph 3	Symbiotic	0.86	.000
	Aposymbiotic	0.56	
Nymph 4	Symbiotic	0.85	.000
	Aposymbiotic	0.51	
Nymph 5	Symbiotic	0.83	.000
	Aposymbiotic	0.46	
Adult	Symbiotic	0.76	.000
	Aposymbiotic	0.40	

### 3.5 Life expectancy

**FIGURE 5:**
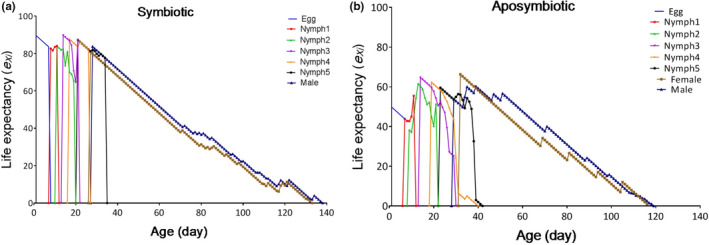
The life expectancy (*e_x_*) of *Graphosoma lineatum* in the symbiotic (a) and the aposymbiotic (b) insects, using the age‐stage, two‐sex life table

The life expectancy (*e_x_*_j_) indicates the length of time that an individual of age *x* and stage *j* is expected to live. The curves revealed that the life expectancy of new hatched eggs of the control and the aposymbiotic insects was estimated to be 88.63 and 48.84 days, respectively (Figure 5a,b).

### 3.6 Life table parameters

The intrinsic rate of increase (*r*) was decreased with decline in symbiont population. Also, the net reproductive rates (*R*_0_) and gross reproductive rates (*GRR*) in the control insects were higher than the aposymbiotics, whereas the mean generation time (*T*) and the finite rate of increase (*λ*) in the control showed no significant difference with the aposymbiotic insects (Table 4).

**TABLE 4:** Population parameters (means ± *SE*) of *Graphosoma lineatum* in the symbiotic and the aposymbiotic insects, calculated by using the age‐stage, two‐sex life table

Treatment/parameters	*GRR* (egg/individual)	*R*_0_ (egg/individual)	*r* (day^−1^)	*λ* (day^−1^)	*T* (day)
Symbiotic	165.692 ± 29.058^a^	115.878 ± 21.624^a^	.080 ± 0.003^a^	1.083 ± 0.004^a^	59.106 ± 0.977^a^
Aposymbiotic	65.649 ± 27.654^b^	20.770 ± 8.992^b^	.045 ± 0.007^b^	1.046 ± 0.024^a^	64.063 ± 4.164^a^

Note

The means followed by the same letter in each row are not significantly different using the paired bootstrap test (*p* < .5). The standard errors were calculated using the bootstrap procedure with 40,000 samples.

## 4 DISCUSSION

Bacterial symbionts play prominent roles in their insect hosts. These symbiotic associations have been described in many of stinkbugs (Duron & Noël, 2016; Hosokawa et al., 2016; Kaiwa et al., 2011; Karamipour et al., 2016; Kikuchi et al., 2009). They harbor their symbionts in the midgut crypts and mostly depend on their symbionts for normal growth and development. Studies on stinkbugs such as Pentatomidae (Bistolas et al., 2015), Plataspidae (Fukatsu & Hosokawa, 2002), Cydnidae (Hosokawa et al., 2012), Acanthosomatidae (Kikuchi et al., 2009), and Scutelleridae (Kafil et al., 2013; Kaiwa et al., 2010; Mehrabadi et al., 2012) have highlighted the importance of bacterial symbiont in normal growth of the host insects. In the present study, we tried to evaluate the relationship between the host insect and the bacterial symbiont by using life table analysis as the life cycle and life history of a species greatly influence its population dynamics and can be used to understand the impact of different biotic and abiotic factors on the biology of insects (Chi & Su, 2006). Since the symbionts are essential in both sexes, the female age‐specific life tables are inadequate. Therefore, we used two‐sex life table to include males in the analysis as well. Among different biological parameters, the intrinsic rate of increase (*r*) is a basic ecological characteristic (Birch, 1948) and the most important parameter in life table analysis (SoumwooD, 1966). This parameter is useful to estimate population growth potential (Ricklefs & Miller, 2000; Roy et al., 2003). In our study, *r* decreased about twofold in the aposymbiotic insects compared to the symbiotics. The results indicated that the symbiotic insects had lower TPOP, because the obtained intrinsic rate of increase (*r*) value for symbiotics was higher than the aposymbiotic insects. Using life table analysis to prove biological impact of symbiont in stinkbugs is rare, nevertheless, it was shown that the bacterial symbiont elimination had negative effects on nymphal development and adult emergence of *Nezara viridula* (Prado et al., 2009), *Brachynema germari*, *Acrosternum heegeri*, and *Acrosternum arabicum* (Kashkouli et al., 2018). Similarly, our results also showed development of different stages in the aposymbiotic insects was longer than the symbiotics.

Despite the first‐instar nymphs of the aposymbiotics showed no difference in terms of behavior and longevity with the symbiotic insects, there were significant differences between aposymbiotics and symbiotics in the other nymphal stages. Furthermore, our data indicated that the rate of emergence of females in the aposymbiotic insects was lower than the symbiotic insects resulting in different sex ratios. These observations suggested that the females are more susceptible to symbiont removal than the males.

We also found significant decrease in other biological factors such as *R*_0_ and *GRR* in the aposymbiotic insects compared with the symbiotics. Moreover, the symbiotic insects had longer life expectancy and age‐stage specific survival rates in comparison with the aposymbiotics. These results clearly showed the biological importance of bacterial symbiont for *G. lineatum*.

Stinkbugs are sucking insects and feed from different parts of the host plant (Schaefer & Panizzi, 2000). Phytophagous diets are limited for essential amino acids and some vitamins (Kenyon et al., 2015). The active feeding stage of *G. lineatum* is the second‐instar nymph; notably, we found that negative effects of symbiont elimination in the aposymbiotic insects occurred in the second instar. The second instar had longer mean duration time and higher mortality in aposymbiotic insects compared with symbiotic insects. Recently, it was shown that *Pantoea carbekii*, the bacterial symbiont of the brown marmorated stink bug, encode the canonical pathway for the biosynthesis of almost all essential and nonessential amino acids (Kenyon et al., 2015). Moreover, we have also found the pathways required for amino acid and vitamin B synthesis in the genome of bacterial symbiont of the pistachio green stink bug that highlights the nutritional importance of this symbiont for the host (Kashkouli, Castelli, et al., 2020). Therefore, it is seems that the symbiont of *G. lineatum* may also provide essential nutrients to support host survival, development, and fecundity. Given that, the elimination of symbiont from *G. lineatum* can make significant changes in insect life history like reduction in female emergence, life expectancy, fecundity, survival rate. For example, reduction in intrinsic rate of increase and female emergence in the absence of symbiont can cause problem in population growth in nature. There is no information regarding nutritional role of maternal anus secretion smear over the egg surface following oviposition in these insects as the first‐instar nymphs are considered as non‐feeding stages; however, we cannot preclude the possibility of taking up nutrients besides symbionts and therefore merit more investigation.

In conclusion, this study confirmed the contribution of the bacterial symbionts in the life history traits of *G. lineatum*. Our study suggested that the PLSGL is necessary for normal development and growth of this insect and must be considered for better understanding of the ecology of this insect. In fact, it seems that the insects harbor the bacterial symbionts to improve their biology.

## CONFLICT OF INTEREST

The authors declare that they have no conflict of interest.

## AUTHOR CONTRIBUTION

**Naeime Karamipour:** Conceptualization (equal); Investigation (equal); Methodology (equal); Software (equal); Visualization (equal); Writing‐original draft (equal). **Yaghoub Fathipour:** Data curation (equal); Methodology (equal); Software (equal); Validation (equal); Writing‐review & editing (equal). **Mohammad Mehrabadi:** Conceptualization (equal); Data curation (equal); Formal analysis (equal); Funding acquisition (equal); Investigation (equal); Methodology (equal); Project administration (equal); Resources (equal); Software (equal); Supervision (equal); Validation (equal); Visualization (equal); Writing‐original draft (equal); Writing‐review & editing (equal).

## Data Availability

The data associated with this publication can be accessed on Dryad (https://doi.org/10.5061/dryad.t4b8gtj12).
